# Applications of Capillary Electrophoresis for the Determination of Cannabinoids in Different Matrices

**DOI:** 10.3390/molecules28020638

**Published:** 2023-01-08

**Authors:** Nicoleta Mirela Blebea, Gabriel Hancu, Robert Alexandru Vlad, Andreea Pricopie

**Affiliations:** 1 Department of Pharmacology and Pharmacotherapy, Faculty of Pharmacy, “Ovidius” University of Constanța, 900470 Constanța, Romania; 2 Pharmaceutical and Therapeutic Chemistry Department, Faculty of Pharmacy, “George Emil Palade” University of Medicine, Pharmacy, Science and Technology of Targu Mures, 540142 Târgu Mures, Romania; 3 Pharmaceutical Technology and Cosmetology Department, Faculty of Pharmacy, “George Emil Palade” University of Medicine, Pharmacy, Science and Technology of Targu Mures, 540142 Târgu Mures, Romania; 4 Biochemistry and Chemistry of Environmental Factors Department, Faculty of Pharmacy, “George Emil Palade” University of Medicine, Pharmacy, Science and Technology of Targu Mures, 540142 Târgu Mures, Romania

**Keywords:** cannabinoids, cannabidiol, tetrahydrocannabinol, capillary electrophoresis

## Abstract

Cannabinoids, terpenophenolic chemicals found only in cannabis, are primarily responsible for cannabis pharmacologic effects; nearly 150 distinct cannabinoids have been identified thus far. Among these, the main psychoactive molecule, tetrahydrocannabinol (THC), and the non-psychoactive counterpart, cannabidiol (CBD) are distinguishable. In the past decade, a CBD-containing pharmaceutical preparation was approved by Food and Drug Administration (FDA) for the treatment of drug-resistant epileptic seizures in children, and research trials for a variety of additional medical conditions for which CBD has been suggested as a therapy are being conducted. Additionally, the number of “CBD-containing” dietary supplements, largely available online, is increasing rapidly. Consequently, the necessity for the development of qualitative and quantitative methodologies for the analysis of the bioactive components of *Cannabis* is rising because of the increase in the production of therapeutic cannabis products. One of the analytical methods with good potential in cannabinoids analysis is capillary electrophoresis (CE). It has advantages related to high separation efficiency, relatively short analysis time, and the small consumption of analytes and reagents which generates relatively lower operational costs than other methods. This review focuses on the use of CE techniques to examine biological matrices and plant materials for the presence of cannabinoids and other bioactive compounds found in cannabis. The advantages, drawbacks, and applicability of the various electromigration approaches are also assessed. The article provides an overview of the “state of the art” and the latest trends in CE-based methods for the determination of cannabinoids.

## 1. Introduction

The *Cannabis sativa* L. plant is annual herbaceous flowering plant species belonging to the *Cannabaceae* family, originally from Central Asia, which has been used for centuries to produce hemp fiber (used for clothing, rope, and paper), seeds (used as food) and as a medicinal plant [1]. *Cannabis* has been utilized since the dawn of human civilization for medicinal purposes and recreational use. *Cannabis* phenotypes are highly variable, and the plant is recognized to have three subspecies: *Cannabis sativa* subsp. *Sativa*, *Cannabis sativa* subsp. *indica*, and *Cannabis sativa* subsp. *ruderalis* [1,2].

*Cannabis* therapeutic ingredients are mainly concentrated in the female flowers of the plant; the so-called “resin” contains a wide range of terpenoids and cannabinoids. *Cannabis*’ medicinal properties are linked mainly to its cannabinoid content and terpenophenolic compounds which are found exclusively in cannabis. To date, almost 150 different cannabinoids have been identified [3]. Among these, we can identify the two major components in the cannabis plant: the main psychoactive compound, tetrahydrocannabinol (THC), and the non-psychoactive compound, cannabidiol (CBD) [3,4]

**Figure 1:**
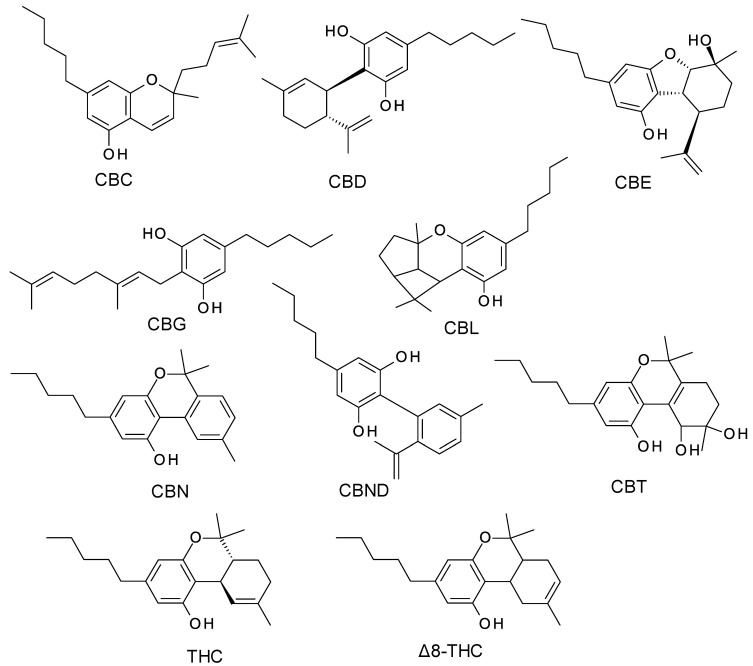
The structure of the most common phytocannabinoids [5].

The term cannabinoid refers to both natural cannabinoids (endocannabinoids; phytocannabinoids) and synthetic cannabinoids that operate on cannabinoid receptors. Phytocannabinoids refer to a group of oxygenated aromatic hydrocarbon metabolites derived from the *Cannabis* plant that contain 21 carbon atoms. Currently, phytocannabinoids are conventionally classified into 11 chemical classes, each named after the “lead” compound: cannabichromene (CBC), cannabidiol (CBD), cannabielsoin (CBE), cannabigerol (CBG), cannabicyclol (CBL), cannabinol (CBN), cannabinodiol (CBND), cannabitriol (CBT), tetrahydrocannabinol (THC), (−)-Δ8-trans-tetrahydrocannabinol (Δ8-THC), and miscellaneous phytocannabinoids (Figure 1) [5]. The existing heterogeneity in phytocannabinoid concentration between and among different chemotypes has significant implications for medical cannabis formulations and administration [6].

Thousands of *Cannabis* strains are currently available on the market with varying phytocannabinoid compositions, which are classified based on the total quantity of THC and CBD. *Cannabis* has gained substantial attention in recent years as an increasing number of countries legalize *Cannabis* for medicinal and recreational use. There is quite a high degree of variation in the amounts of THC allowed by various legislation in hemp preparations, ranging from 0.05 to 0.5%. The legal status of *Cannabis’s* main components varies from country to country, with some countries classifying THC and CBD in the same class of illegal narcotics and others legalizing CBD products [7,8].

**Figure 2:**
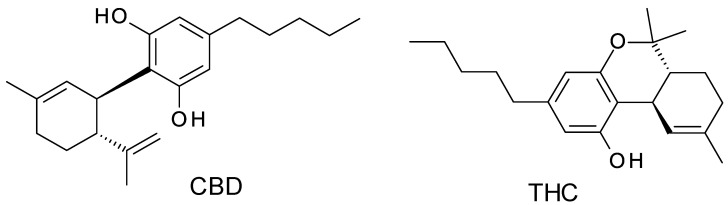
Chemical structures of CBD (2-[(1*R*,6*R*)-3-methyl-6-prop-1-en-2-ylcyclohex-2-en-1-yl]-5-pentylbenzene-1,3-diol) and THC ((6a*R*,10a*R*)-6,6,9-trimethyl-3-pentyl-6a,7,8,10a-tetrahydrobenzo[c]chromen-1-ol).

While CBD is chemically related to THC (the difference is that CBD is a bicyclic while THC is a tricyclic compound, but the molecular mass is the same) (Figure 2), CBD has shown significant tolerance in humans with limited abuse potential. CBD’s good safety profile has resulted in the recent reduction of legal and regulatory restrictions, made CBD products available in numerous countries, and led to a surge in interest in CBD treatment [9]. This led to the situation that CBD therapeutic demand has outpaced scientific research and regulatory development, creating a complex ecosystem of disinformation and dubious health claims.

Currently, there are two phytocannabinoid pharmaceutical preparations approved by the USA Food and Drug Administration (FDA): Epidiolex^®^—oral solution (contains only CBD) and Sativex^®^—oromucosal spray (contains both CBD and THC). Epidiolex^®^ is a medication used to treat seizures in Lennox-Gastaut or Dravet syndrome in individuals aged 2 years and older [10,11]. Because of their popularity, various CBD-containing dietary supplements can be found on the market. Furthermore, CBD can be found in essential oils, personal care products, foods, and medicinal formulations.

CBD is a versatile substance in terms of the pathologies it can treat and for which it could be administered as adjuvant treatment, among these we can mention epilepsy, anxiety, neuropathic pain, or cancer [12].

Cannabinoids are lipophilic and can be rapidly absorbed; much of the available pharmacokinetic data focus on CBD and THC. CBD and THC pharmacokinetic profiles differ significantly between users, dosage and form, acute and chronic usage, and mode of administration. Smoking and vaporizing cannabis results in higher blood levels of cannabinoids, a faster start of the effect, and more bioavailability by comparison to oral ingestion [13].

Whereas THC is a partial agonist of the cannabinoid receptors (CB1 and CB2) in the endogenous cannabinoid system and exerts its psychoactive and pain modulatory effects via CB1 agonist action, CBD has relatively little affinity for the orthostatic sites of these receptors and may even inhibit THC binding at CB1 receptors via another mechanism. Cannabinoids have a wide range of effects via the activation of G-protein-coupled cannabinoid receptors in the brain and peripheral organs. CBD has also been demonstrated to bind to non-cannabinoid receptors [13,14].

Synthetic cannabinoids are a class of “designer drugs” that binds to the same receptors to which endocannabinoids attach. Synthetic cannabinoids are becoming a major public health problem due to their rising usage as well as their unpredictable toxicity and misuse potential. Synthetic cannabinoids are linked to greater rates of toxicity and hospitalization than natural cannabis, most likely because they are direct agonists of cannabinoid receptors, whereas THC is a partial agonist [15].

Due to the limited means of consumers to analyze the chemical composition of *Cannabis* products, people may unwittingly purchase items with low quality, especially in regard to dietary supplements acquired online [16]. As *Cannabis* product usage becomes more widely acknowledged, it becomes increasingly vital to evaluate the cannabinoid profile and quantity of cannabis products to assure consistency and quality of the products. Developing effective analytical methods for the determination of CBD and other related cannabinoids is a major issue in pharmaceutical and biological research because of its potential use as a medication or as a component of dietary supplements.

Several reviews regarding the analysis methods applied for the determination of cannabinoids from different matrices have been published in the last 15 years; among these, we can distinguish those by Presley [17], Raharjo and Verpoorte [18], Ramirez et al. [19], Pourseyed Lazarjani et al. [20] and Micalizzi et al. [21]. Most of the methods used for the determination of cannabinoids are chromatographic ones, especially high-performance liquid chromatography (HPLC) and gas chromatography (GC). For the analysis of CBD and related compounds, HPLC combined with diode-array (UV/DAD) or mass spectrometry (MS) detection is without a doubt the most widely used analytical technique. However, the tendency for the application of novel analytical approaches for the quantitation of CBD and related compounds in plant material and other derived products, such as nuclear magnetic resonance spectroscopy (NMR) and near-infrared spectroscopy (NIR), can be explained by the growing need for faster, more automated, and environmentally friendly methodologies [22,23].

Capillary electrophoresis (CE) techniques are considered an alternative and a complementary method for HPLC techniques, with advantages related to high separation efficiency, relatively short analysis time, and especially low consumption of analytes and reagents which generates lower operational cost. Additionally, because usually in CE low amounts of organic solvents are used, this technique folds very well on the concept of “green chemistry” [24].

Several CE applications for the determination of cannabinoids in different matrices have been published in the last 20 years, but to our knowledge, no review regarding the use of electromigration techniques in the analysis of cannabinoids has been published so far.

This review focuses on the CE-based analytical methods employed to analyze both plant materials and biological matrices concerning both cannabinoid content and other bioactive substances contained in cannabis. To provide helpful recommendations for the selection of the most appropriate electromigration method for the analysis of cannabinoids in either biological or plant samples, essential issues are discussed. The advantages, drawbacks, and applicability of the various electromigration approaches are also assessed.

## 2. CE Methods for the Determination of CBD and Related Substances

### 2.1. Non-Aqueous Capillary Electrophoresis (NACE)

NACE is a kind of capillary zone electrophoresis (CZE) in which ionic solutions in organic solvents are used as a background electrolyte (BGE). The major advantage of utilizing organic solvents instead of water in CE is an improvement in the solubility of hydrophobic analytes, like cannabinoids. The NACE solvent, whether a single organic solvent or a mixture, should be able not only to dissolve the hydrophobic analytes but also BGE to create an acceptable pH and conduct current when an electric field is applied. By comparison with classic CZE, NACE provides different interaction possibilities, such as ion pairing, and homo- or heteroassociation, which may result in intriguing changes in separation selectivity. Furthermore, the use of organic solvents may result in shorter migration times and improved separation efficiencies, as separation parameters may be managed on a larger scale with organic solvents than with water, consequently, high variations in electrophoretic mobilities of the analytes can be accomplished and separations that are not possible in aqueous CE can be carried out with superior selectivity because solvent characteristics will affect the analytes’ acid-base behavior [25,26].

A NACE with electrochemical detection (NACE–ED) was applied for the determination of cannabinoids in hair by Backofen et al. Different BGE compositions were tested for the separation of CBD, CBN, THC, and THC carboxylic acid (THCA). The best results were obtained when using a strongly basic BGE composed of 5 mM sodium hydroxide dissolved in an acetonitrile–methanol mixture (1:1). In the oxidation mode, electrochemical detection produced well-defined signals. Since the analysis of cannabinoids in a biological matrix requires low limits of detection (LODs), experimental settings that affect sensitivity and noise level were optimized. LOD for THC was 37 ng/mL, which is about two orders of magnitude lower by comparison with on-column UV detection. Proper sample preparation was essential for the determination of cannabinoids in hair, solid-phase extraction (SPE) utilizing a patented sorbent reduced matrix interference considerably more successfully than liquid–liquid extraction (LLE) [27].

Iwamuro et al. published a NACE-MS method for the determination of THC, its metabolite 11-nor-9-carboxy-Δ8-THC (THC-COOH), and its glucuronide (THC-glucuronide) in urine. The BGE contained 40 mM ammonium formate at pH 6.4. Migration time was less than 10 min, and the entire analytical process, including sample preparation, took around 30 min. The only pretreatment required for urine samples was dilution with methanol and centrifugation to achieve LODs of 50 ng/mL due to the use of a highly selective detector, such as MS. The use of multiple reaction mode (MRM) improved the method’s selectivity and specificity. The method was applied for the cannabinoid’s determination in urine samples from cannabis users [28].

A NACE method with light-emitting diode induced fluorescence (LEDIF) detection for the determination of CBD and THC in oral fluids was developed by Mazina et al. Salivette^®^ sampling equipment was used for the collection, preparation, and pre-concentration of the saliva sample into a single process. The BGE contained 2.5 mM sodium hydroxide dissolved in methanol: acetonitrile mixture (1:1). Separation time was less than 6 min; CBD migrated faster than THC. LOD values were 0.17 and 0.19 µg/mL for CBD and THC, respectively. LEDIF was used for detection, with 280 nm excitation and 307 nm emission. The applicability of the method was tested on oral fluid samples after controlled smoking of a marijuana cigarette. Although the CE-LEDIF method’s LODs are higher than in GC-MS, they can nevertheless be deemed appropriate for measuring cannabinoids in oral fluids shortly after smoking (a couple of hours) [29].

A NACE method coupled with a fluorescence detector (FD) was developed by Saar-Reismaa for assessing in situ the usage of illicit substances in oral fluids including cannabinoids (CBD, THC) together with several amphetamine derivatives. The portable CE-FD using the developed electrophoretic techniques with a 230–255 nm excitation wavelength range was effectively used for the detection of illicit drug misuse at a music festival in Estonia. The BGE for the determination of cannabinoids contained 5 mM sodium hydroxide dissolved in a methanol: acetonitrile mixture (1:1). The LOD and LOQ for both cannabinoids were 25 µg/L and 83 µg/L, respectively. For the determination of amphetamines, amines which can be ionized in an acidic BGE, the determination was made by CZE using a different BGE composed of 42.5 mM phosphoric acid and 30 mM TRIS at pH 2.5. This study demonstrates the applicability of deep UV excited fluorescence detection for portable CE instruments for the identification of drug abuse by analyzing the oral fluid of suspects in situ [30].

**Figure 3:**
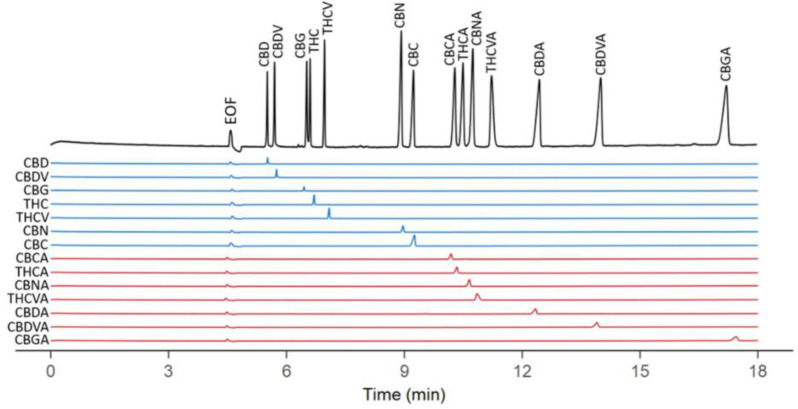
CE electropherograms of 14 cannabinoids in a standard mixture and individually (electrophoretic conditions: 6.5 mM sodium hydroxide in acetonitrile:water (60:40) mixture, 25 mM µM β-CD, 45 V/cm, UV detection 230 nm, sample concentration 50 µg/mL; Blue electropherograms—decarboxylated forms of cannabinoids (CBD—cannabidiol, CBDV—cannabidivarin, CBG—cannabigerol, THC—tetrahydrocannabinol, THCV—tetrahydrocannabivarin, CBN—cannabinol, CBC—cannabichromene), red electropherograms—carboxylated forms of cannabinoids (CBCA, THCA, CBNA, THCVA, CBDA, CBDVA, and CBGA)). The figure is reproduced from Zaripov et al. [31] with permission from MDPI.

Quantification of 14 cannabinoids (including CBD and THC, decarboxylated and acidic forms of cannabinoids) from extracted cannabis samples was carried out by Zaripov et al. by NACE with UV detection using an acetonitrile-based BGE in the presence of β-cyclodextrin (β-CD). By comparison with the methods mentioned earlier, this method uses a partially aqueous BGE. Because the CD is not soluble in the absence of water, the use of a completely non-aqueous BGE was not possible in this case. The addition of β-CD in the BGE provides orthogonal separation media by transiently interacting with the analytes based on their shape and polarity. While separation of these 14 cannabinoids was achievable without CD, its addition reduces analysis time by 10 min, resulting in higher resolution due to reduced longitudinal diffusion. The best results were obtained when using a 6.5 mM sodium hydroxide in 60% acetonitrile BGE at pH 12.0 and 25 µM β-CD as a buffer additive. The separation time was approximately 18 min. Decarboxylated cannabinoids migrated before the acidic forms, due to the higher overall negative charge of acidic cannabinoids due to their carboxyl group (Figure 3). The LODs were between 1.2–1.8 µg/mL. The CE results were compared with the ones obtained with a HPLC method, and comparable separation and running times were obtained, however, the HPLC approach did not completely resolve several compounds. On a C18 stationary phase, cannabinoids are difficult to separate because of their highly similar structural characteristics and polarities; better separation might be achieved by decreasing the column’s particle size and increasing pressure, or by using a UHPLC system. A disadvantage of this CE method is the low buffering capacity of BGE, as acetonitrile from the BGE evaporates quickly after the vial is opened (fresh BGE should be used for every determination), resulting in pH fluctuations. Taking into consideration these disadvantages the reproducibility of this method is rather low [31].

### 2.2. Micellar Electrokinetic Chromatography (MEKC)

MEKC is a modified version of CE, in which charged surfactants are added to the BGE at a concentration above their critical micellar concentration (CMC) allowing the separation of neutral or charged analytes as a function of their affinity to partition into micelles. MEKC extends the application of CE techniques for the analysis of neutral compounds which cannot be separated through the classic CZE, demonstrating a remarkable ability to deal with complex biological and non-biological matrices. MEKC is a useful option for the analysis of cannabinoids which, due to their hydrophobic nature and their lack of own electrophoretic mobility, will co-migrate with the electroosmotic flow (EOF) in aqueous CZE resulting in unresolved peaks [32,33].

The first article regarding the application of MEKC for the detection of cannabinoids was published by Weinberger and Lurie in 1991. In this study, a large number of illicit substances were analyzed by MEKC, including CBD and THC, using a BGE composed of 8.5 mM borax, 8.5 mM disodium hydrogenophosphate, 85 mM sodium dodecyl sulfate (SDS), and 15% acetonitrile at a pH 8.5. However, this was an article about the general applications of MEKC in the screening of illicit drug substances [34].

In 1992 Wernly and Thormann applied a MEKC method for the determination of THC main metabolite THC-COOH in urine. A BGE containing 6 mM borax and 10 mM disodium hydrogenophosphate and 75 mM SDS at pH 9.1 was used in the determination. Concentrations as low as 10 ng/mL THC-COOH were accurately measured [35].

**Figure 4:**
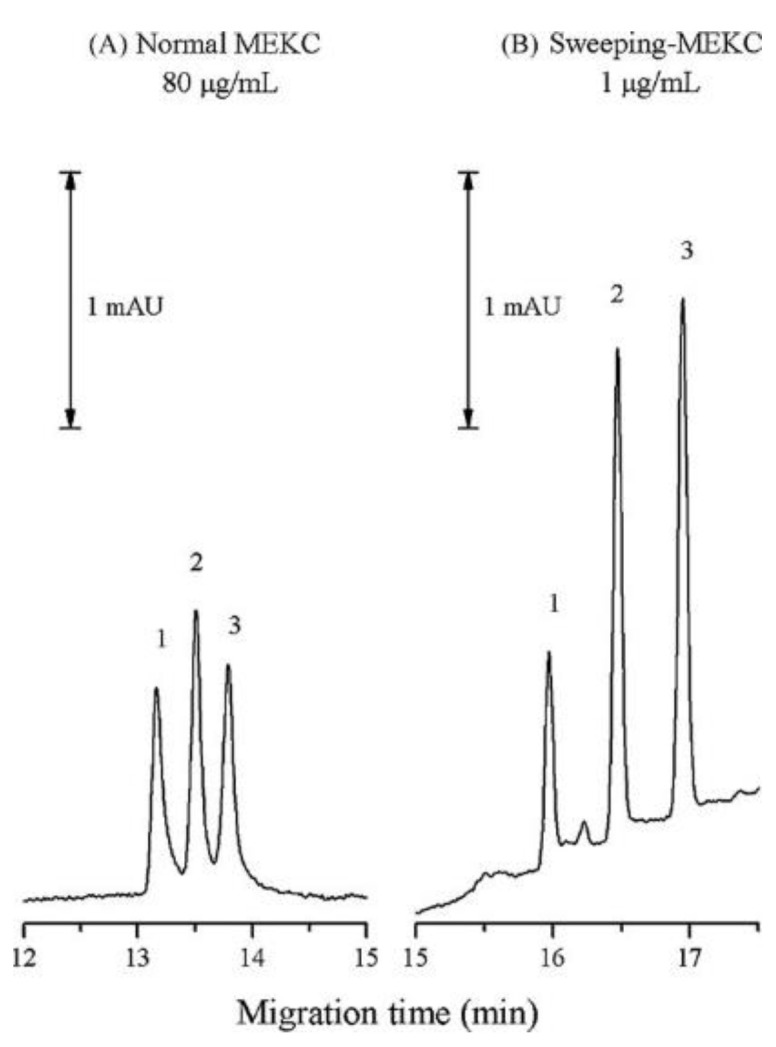
Comparison between the normal MEKC and sweeping-MEKC methods for simultaneous separation of THC and its metabolites (analytical conditions (**A**)—25mM citric acid/disodium hydrogenphosphate BGE, 75 mM SDS, pH 2.6, 40% methanol, 25 °C, −20 kV, injection 3.45 kPa × 3 s, sample concentration, 80 µg/mL; (**B**)—25mM citric acid/disodium hydrogenphosphate BGE, 75 mM SDS, pH 2.6, 40% methanol, 25 °C, −20 kV, injection 3.45 kPa × 300 s, sample concentration, 1 µg/mL). Peaks: (1) THC; (2) THC-COOH; (3) THC-OH. The figure is reproduced from Su et al. [36] with permission from Elsevier.

Su et al. developed a sensitive MEKC approach for the simultaneous measurement of THC and its metabolites (11-hydroxy-Δ9-THC-THC-OH, THC-COOH) in urine. In comparison to traditional MEKC with UV detection, the authors’ use of SPE for sample clean-up and off-line preconcentration as well as on-line preconcentration based on sweeping led to enhancement factors of up to 200-fold higher. The effects of several sweeping-MEKC analytical parameters on the separation were tested in a univariate mode. A comparison between the results of normal MEKC and sweeping-MEKC methods is presented in Figure 4. The best results were obtained when using a BGE composed of 25 mM citric acid/disodium hydrogenphosphate, 75 mM SDS and 40% methanol (*v/v*) at pH 2.6. The LODs ranged from 3.87 to 15.2 ng/mL (below the cut-off levels in urine of 50 ng/mL). The total analysis time including sample preparation was approximately 80 min, which makes the method less appealing for routine analysis. The sweeping MEKC method proved to be a useful tool for determining, with high sensitivity, THC, and its metabolites in the urine of suspected THC users [36].

Gottardo et al. applied a MEKC with DAD detection for the determination of synthetic cannabinoids in herbal blends, which could be present in “spice” products. The optimized BGE contained 25 mM borax, 30 mM SDS and 20% n-propanol (*v/v*) at pH 8.0. The optimization of analytical conditions was made using a univariate approach, by varying one parameter while maintaining the others constant. Without any appreciable matrix interference, baseline separation was achieved under optimized conditions in approximately 25 min. The LODs ranged between 1–1.5 µg/mL. The method was capable to separate 10 cannabinoids in a single run. The technique, needing just sample dilution, was effectively used to identify synthetic cannabinoids in 15 distinct herbal mix samples. The herbal blends that were examined showed a wide range in the amounts of synthetic cannabinoids they contained, ranging from 1.6 to 28.1%. Additionally, the developed MEKC separation was used to calculate log P (octanol/water partition coefficients) of the substances, used as a measure of lipophilicity [37].

Akamatsu and Mitsuhashi described a MEKC-MS/MS method for the simultaneous determination of 12 synthetic cannabinoids in illegal herbal blends. The BGE consisted of 50 mM ammonium perfluorooctanoate in 20% acetonitrile solution at pH 9.0. The LOD values were between 6.5–7.65 µg/g. To achieve accurate identification, it was efficient to separate analytes in the migration time as well as the transition by MRM. The proposed method was applied for the identification of synthetic cannabinoids in herbal blends [38]

Cheng et al. developed a MEKC method for monitoring THC and its metabolites (THC-OH, THC-COOH) in urine samples. To increase analytical sensitivity, a new on-line preconcentration CE technique integrating large volume sample injection, anion selective exhaustive injection, and sweeping was developed. The optimum BGE contained 30 mM phosphate, 100 mM SDS, and 40% methanol (*v/v*) at pH 2.5. With sample pretreatments, this method’s overall duration was around 2 h. Up to a 2000-fold boost in sensitivity was achieved under optimized circumstances. The intricate derivatization processes do not need to be addressed, and the detection sensitivity was comparable with MS detection. The LODs were 10 ng/mL for THC, 5 ng/mL for THC-OH, and 0.5 ng/mL for THC-COOH [39].

Because of the presence of SDS in the BGE, the classic MEKC method has little potential for being used with MS detection, not being compatible with electrospray ionization (ESI). However, Švidrnoch et al. employed perfluoroheptanoic acid as a volatile micellar phase in the BGE for the separation and identification of 15 chosen naphthoyl- and phenylacetylindole-derived synthetic cannabinoids using MEKC-MS. The MEKC separation and MS detection are made possible by perfluorinated surfactants without ion source contamination or signal suppression. The effect of perfluoroheptanoic acid concentration in the BGE on MS detection and separation was investigated. A BGE containing 150 mM ammonium hydroxide, 75 mM perfluoroheptanoic acid, and 10% isopropanol (*v/v*) at pH 9.2 was used. The LODs were between 0.9–3.0 ng/mL. To demonstrate the method’s potential for use in forensic and toxicological determinations, it was used for the separation and identification of investigated analytes following LLE in spiked urine and serum samples [40].

### 2.3. Capillary Electrochromatography (CEC)

CEC is a “hybrid” technique that combines the best characteristics of CE (i.e., separation efficiency) with the best characteristics of HPLC (i.e., well-characterized retention and selectivity mechanisms, ability to handle highly hydrophobic compounds, increased sample capacity). Electrically driven reversed-phase (RP)-CEC has the potential to achieve 5–10 times the efficiencies of RP-HPLC because of EOF, which produces a plug like velocity profile while transporting the mobile phase and solutes in CEC [41].

**Figure 5:**
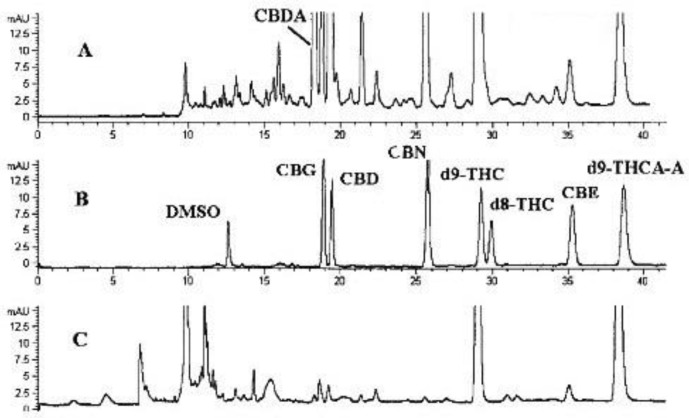
CEC separation of cannabinoids in (**A**)—concentrated hashish extract, (**B**)—standard mixture, (**C**)—concentrated marijuana extract (analytical conditions: 3-µm CEC Hypersil C18 capillary, 25 mM phosphate in acetonitrile, pH 2.57, 20 °C, 25 kV, electrokinetic injection 5 kVx 3 sec, UV detection 210 nm) (DMSO—dimethyl sulfoxide, CBG—cannabigerol; CBD—cannabidiol; CBN—cannabinol; Δ9-THC—tetrahydrocannabinol; Δ8-THC—(−)-Δ8-trans-tetrahydrocannabinol; CBE—cannabielsoin; Δ9-THC-A—tetrahydrocannabinol acid). The figure is reproduced from Lurie et al. [42] with permission from American Chemical Society.

Lurie et al. applied a CEC method with UV detection for the determination of seven cannabinoids (CBC, CBD, CBG, CBN, THC, Δ8-THC, ∆-9-tetrahydrocannabinolic acid). Separation was achieved using a 3-µm CEC Hypersil C18 capillary with an acetonitrile/phosphate (pH 2.57) mobile phase. The effects of analytical parameters on the separation were verified by applying a univariate strategy. Using a high-sensitivity UV flow cell with an extended path length of 1.2 mm, LODs approaching the ones obtained by HPLC were obtained. The method was applied to concentrated extracts of hashish and marijuana from drug seizures (Figure 5) [42].

Applications of CE techniques for the determination of cannabinoids are summarized in Table 1. The articles are listed in the table chronologically.

## 3. Conclusions

The analytical scenario surrounding cannabinoids determination is extremely diverse comprising different analytical methods, as no standardization on the criteria for the determination of CBD, THC and related compounds in the plant, plant-derived products, and biological matrices has been established thus far. The identification and detection of a wide range of cannabinoids in various types of matrices has become a difficult challenge for analytical chemists. Taking into consideration these aspects, the purpose of this study is to provide an overview of the most recent achievements in the field of cannabinoids analysis and bioanalysis by CE.

Analyzing the number of published CE studies for the determination of cannabinoids, we conclude that are only a few, considering that the method has greater potential to be used in this area.

CE is an alternative separation technique for the more frequently used chromatographic ones (HPLC, GC) based on the intrinsic charges of the analytes and relies on their different electrophoretic mobilities to separate them inside a narrow silica capillary. The benefits of CE are widely established and are mostly related to the speed of analysis, separation efficiency, and the minimal consumption of analytes and reagents which will generate relatively smaller operational costs [43]. However, one must consider the CE detection limits, which are often several orders of magnitude larger than those of conventional chromatographic and spectroscopic methods. The discovery of techniques to boost CE sensitivity has therefore become a critical subject, with several approaches described. More adequate LOD can be obtained by using, if possible, fluorescence or MS detection instead of classic UV detection [44].

The lack of sensitivity has been addressed through a variety of methods, including the use of chromatographic and electrophoretic preconcentration techniques. These stages, however, have certain drawbacks, such as more sophisticated and time-consuming procedures and reduced reproducibility [45]. Future advancements in this area should center on the development and use of more complex and efficient on-line preconcentration procedures to obtain superior analytical performance.

Cannabinoids have very similar chemical structures, and consequently, similar charges and electrophoretic mobilities; therefore, separation by CZE is difficult to achieve, resulting in overlapping peaks, migration together with the EOF, or excessively long separation times.

Cannabinoids are hydrophobic substances, poorly soluble in water, requiring the presence of an organic solvent (acetonitrile, methanol) in the BGE to optimize solubility and separation. Taking into consideration these aspects, NACE could be an optimal solution for cannabinoids determination by CE. NACE involves the separation of analytes in a medium composed of organic solvents. Changes in separation selectivity in NACE contribute to better separation of certain chemicals with extremely modest charge-to-mass differences in aqueous phases [46].

Another approach exploiting the hydrophobic nature of cannabinoids is the use of MEKC with SDS as a surfactant, which is a suitable separation method for both neutral and ionized substances. MEKC is ideal for the separation of neutral chemicals, such as cannabinoids, and has a high capacity to cope with complicated biological and non-biological matrices. Employing stacking in SDS presence to suppress EOF can result in increased sensitivity, overcoming one of CE’s greatest problems [47]. However, the analysis of neutral substances by MEKC-MS has a significant disadvantage because commonly employed surfactants, such as SDS, are nonvolatile and can induce analyte signal suppression and spectrometer contamination. When connected to MS, a partial filling strategy of MEKC must be employed to minimize ion suppression owing to the presence of surfactants in the ESI and to reduce MS contamination. This problem can be also resolved using so-called “MS-friendly” surfactants like perfluorooctanoic acid [48].

An important issue is also the detection of synthetic cannabinoids; these substances are purposely added in herbal blends and have significant affinities for cannabinoid receptors; some synthetic cannabinoids are more potent than the main psychoactive ingredient in cannabis (THC). Therefore, the development of simple screening approaches is crucial to identify these cannabinoids. The hyphenation of CE with MS offers high sensitivity and the possibility of structurally characterizing analytes, like “spiced” synthetic cannabinoids.

Most of the available research focuses on identifying cannabinoids on biological samples, with urine being the most used matrix. Considering this, additional effort is required to create CE-based methods suitable for assessing additional biological fluids and other various matrix types.

Due to the hydrophobic nature of cannabinoids, CE techniques are not the first-choice methods for their determination, and in this case, LC-MS might be preferable to be used.

Relatively few of the reported publications offer thorough method validation in accordance with generally recognized worldwide regulations, which is a disadvantage because it has a detrimental influence on the methods’ reliability. The next challenge for researchers will be to design CE-based processes that allow method application with more sensitivity, accuracy, and reliability than is typically attained using chromatographic techniques.

## Figures and Tables

**Table 1:** CE methods for the determination of cannabinoids.

CE Technique	Analyte	Matrices	Analytical Conditions	References
CEC-UV	CBC, CBD, CBG, CBN, THC, Δ8-THC, THCA	plant extract	25 mM phosphate in acetonitrile, pH 2.57, 20 °C, 25 kV, electrokinetic injection 5 kV × 3 s Hypersil C18 3-µm CEC capillary	[42]
NACE-ED	CBD, CBN, THC, THCA	hair	5 mM sodium hydroxide dissolved in acetonitrile-methanol (1:1), 20 kV, 220 nm	[27]
MEKC-UV	THC, THC-OH, THC-COOH	urine	25 mM phosphate, 75 mM SDS, 40% methanol (*v/v*), pH 2.6, 25 °C, −20 kV, UV 210 nm	[36]
MEKC-UV	9 synthetic cannabinoids	herbal blends	25 mM borax, 30 mM SDS, 20% n-propanol (*v/v*), pH 8.0, 25 °C, 30 kV, UV 220 nm	[37]
NACE-MS	THC, THC-COOH, THC-glucuronide	urine	40 mM ammonium formate, pH 6.4, 25 °C, 30 kV MS: gas temperature, 300 °C; gas flow (N_2_) 8 L/min; nebulizer, N_2_ 10 psi at electrophoresis, 0 psi at sample injection. capillary 4000 V, sheath liquid 5mM ammonium formate:methanol (50/50, *v/v*)	[28]
MEKC-MS	12 synthetic cannabinoids	herbal blends	50 mM perfluooctanoic acid, 20% acetonitrile/water (*v/v*), 25 °C, 30 kV MS: nebulizing and drying gas 69 kPa N_2_ and 10 L/min N_2_ at 30 °C, sheath liquid 5 mM ammonium formate in 50% *v/v* methanol/water	[38]
NACE-LEDIF	CBD, THC	saliva	2.5 mM sodium hydroxide in methanol-acetonitrile (1:1), 17 kV, λex/em = 280/305 nm	[29]
MEKC-UV	THC, THC-OH, THC-COOH	urine	30 mM phosphate, 100 mM SDS, 40% methanol (*v/v*), pH 2.5, 25 °C, 30 kV, UV 214 nm	[39]
MEKC-MS	synthetic cannabinoids	spiked urine spiked plasma	150 mM ammonium hydroxide, 75 mM perfluoroheptanoic acid, 10% isopropanol (*v/v*), pH 9.20, 25 °C MS: ESI voltage +3.5 kV; drying gas (N_2_) flow rate 10 L/min, temperature 200 °C, nebulizing gas pressure 5 psi, sheath liquid methanol: water: formic acid (50:49.5:0.5, *v/v/v*)	[40]
NACE-UV	14 phytocannabinoids (including CBD, THC)	plant extract	6 mM sodium hydroxide dissolved in acetonitrile:water (60:40), 25 µM β-CD, 450V/cm, UV 230 nm	[31]

## Data Availability

Not applicable.

## Abbreviations

BGE—background electrolyte; β-CD—β-cyclodextrin; CBC—cannabichromene; CBD—cannabidiol; CBE—cannabielsoin; CBG—cannabigerol; CBL—cannabicyclol; CBN—cannabinol; CBND—cannabinodiol; CBT—cannabitriol; CE—capillary electrophoresis; CEC—capillary electrochromatography; CMC—critical micellar concentration; CZE—capillary zone electrophoresis; DAD—diode array detection; ED—electrochemical detection; EOF—electro osmotic flow; ESI—electrospray ionization; FDA—Food and Drug Administration; GC—gas chromatography; HPLC—high performance liquid chromatography; LEDIF—light-emitting diode induced fluorescence; LLE—liquid-liquid extraction; LOD—limit of detection; MEKC—micellar electrokinetic chromatography; MRM—multiple reaction mode; MS—mass spectrometry; NACE—non-aqueous capillary electrophoresis; NIR—near infrared spectroscopy; NMR—nuclear magnetic resonance spectroscopy; RP—reverse phase; SDS—sodium dodecyl sulfate; SPE—solid-phase extraction; THC—tetrahydrocannabinol; Δ8-THC—(−)-Δ8-trans-tetrahydrocannabinol; THC-OH—11-hydroxy-Δ9-THC; THC-COOH—11-nor-9-carboxy-Δ8-THC.
